# B7-H3-Induced Signaling in Lung Adenocarcinoma Cell Lines with Divergent Epidermal Growth Factor Receptor Mutation Patterns

**DOI:** 10.1155/2020/8824805

**Published:** 2020-12-24

**Authors:** Meng Ding, Haixiu Liao, Nannan Zhou, Ying Yang, Shihe Guan, Liwen Chen

**Affiliations:** Department of Laboratory Medicine, Second Hospital of Anhui Medical University, 678 Furong Road, Hefei, Anhui 230601, China

## Abstract

The cosignal molecule B7-H3 is gaining attention due to its abnormal expression and abundant signal transduction in many types of malignancies. B7-H3-induced signaling includes at least three cascades: PI3K/AKT, JAK2/STAT3, and Raf/MEK/ERK1/2, which are also involved in epidermal growth factor receptor- (EGFR-) triggered signaling in lung adenocarcinoma cells. However, the correlation between B7-H3-induced signaling and EGFR signaling, and between B7-H3-targeted immunotherapy and EGFR-targeted therapy in lung adenocarcinoma, remains to be elucidated. Herein we find that knockout of *B7-H3* gene decreased cell survival and increased EGFR-tyrosine kinase inhibitor gefitinib susceptibility of both H3255 and HCC827 cells, two lung adenocarcinoma cell lines harboring EGFR L858R (exon 21) and Del E746-A750 (exon 19) mutations, respectively. *B7-H3* deletion resulted in dramatic reduction of phosphorylation level of AKT and STAT3 in H3255 cells while having mild-to-moderate suppression on AKT, STAT3, and ERK1/2 in HCC827 cells. Gefitinib had similar effects with *B7-H3* deletion both in H3255 and HCC827 cells. Furthermore, *B7-H3* ablation had significant synergistic effects with gefitinib in HCC827 cells. Collectively, our study reveals B7-H3-induced signaling in lung adenocarcinoma cell lines with divergent EGFR mutations, and a translational potential of combined targeted therapy of B7-H3 and EGFR in lung adenocarcinoma with EGFR Del E746-A750 mutation.

## 1. Introduction

Lung cancer ranks first for both incidence and mortality upon 2018 worldwide cancer statistics, with a percentage of 11.6% of the total cases and 18.4% of the total cancer deaths, respectively [1]. An overwhelming percentage of lung cancer (80-85%) is non-small cell lung cancer (NSCLC), among which approximately half is adenocarcinoma [2, 3]. In recent years, tumor genetics was included into subtype identification, represented by the mutation status of epidermal growth factor receptor (*EGFR*) in lung adenocarcinoma [3, 4].

EGFR signaling is transduced by recruiting the PI3K/PKB (AKT), JAK/STAT3, and Raf/MEK/ERK1/2 pathways [5]. Activating somatic mutations in the tyrosine kinase domain of *EGFR* gene are prevalent in lung adenocarcinoma, which are associated with the clinical response to EGFR-tyrosine kinase inhibitors (TKIs) [6, 7]. Two most frequent mutations, in-frame deletions in exon 19 (Del E746-A750) and a point mutation in exon 21 that substitutes an arginine for a leucine at codon 858 (L858R), constitute nearly 90% of all EGFR mutations [8].

Being a type I transmembrane glycoprotein of the B7 superfamily, B7-H3 (CD276) is abnormally upregulated in series of solid tumors including NSCLC [9–12]. An unknown B7-H3 ligand was detected on activated T cells; however, the role of B7-H3 in T cell immune regulation is still under debate [13–16]. In contrast, numerous studies have unanimously shown that B7-H3 plays a negative role in cancer progression. B7-H3 triggers protumorigenic signals including PI3K/AKT, JAK2/STAT3, and Raf/MEK/ERK1/2 to promote cancer invasion, migration, angiogenesis, drugs sensitivity, and the Warburg effect [17–24]. Thus, B7-H3 may act as a new target for cancer immunotherapy [19–25]. B7-H3-induced signaling and EGFR signaling have similar pathways; however, the correlation between the two signaling cascades remains to be elucidated. Herein, we analyze B7-H3-induced signaling and its potential correlation with EGFR signaling in lung adenocarcinoma H3255 (L858R) and HCC827 (Del E746-A750) cells and explore the promising combination of B7-H3-targeted immunotherapy and EGFR-targeted therapy in lung adenocarcinoma.

## 2. Materials and Methods

### 2.1. Reagents and Media

The RPMI 1640 media was obtained from Invitrogen (Gibco BRL, Grand Island, NY, USA). WST-8/CCK-8 was purchased from Dojindo (Kumamoto, Japan). Camptothecin (CPT) was from Sigma-Aldrich (St. Louis, MO, USA). Gefitinib was from MCE (MedChem Express, New Jersey, USA).

### 2.2. Cell Lines and Cell Culture

Lung adenocarcinoma cell lines H3255 and HCC827 were from Cell Culture Center of Fuheng Biology (Shanghai, China), cultured in RPMI 1640 supplemented with 10% FBS (Lonsera Science SRL) and penicillin (100 IU/mL)/streptomycin (100 *μ*g/mL). Both cell lines were authenticated by using short tandem repeat (STR) analysis in combination with sex-typing gene amelogenin detection and compared with DSMZ STR cell line profiles before use.

### 2.3. Genome Editing of B7-H3 Using the CRISPR/Cas9 System

Knockout (KO) of *B7-H3* gene in H3255 and HCC827 cell lines was conducted using CRISPR/Cas9 guide constructs based on a previously published protocol [26]. Briefly, by means of a bulge-allowed quick guide-RNA designer (http://rgenome.net/cas-designer/), sequences for single guide-RNA (sgRNA) were preferentially chosen within the exon 4 of *B7-H3* genomic gene. The target sequences of sgRNA were used as follows: 5'-caccgTTGATGTGCACAGCGTCCTG-3' (forward) and 5'-aaacCAGGACGCTGTGCA CATCAAc-3' (reverse). The potential off-target numbers within 2nt mismatches and out-of-frame scores <66 were excluded to limit CRISPR off-targets. The lentivirus expressing only Cas9 was used to generate negative control (mock) cells.

### 2.4. Cell Proliferation and Cell Growth Suppression Analysis

*B7-H3* KO and mock H3255 and HCC827 cells were cultured for 0 h, 24 h, 48 h, and 72 h, and the fold change of cell proliferation was assayed by quantitation of the uptake and digestion of WST-8/CCK-8 according to the manufacturer's instructions (Dojindo Laboratories). Growth-suppressive effects were measured in *B7-H3* KO and mock H3255 and HCC827 cells treated by gefitinib. The cell viability was determined by WST-8/CCK-8, and the positive control group (cells left untreated) was normalized to 1.0. Cell survival rate = (OD value of treatment group − OD value of blank control group)/(OD value of negative control group − OD value of blank control group). All experiments were performed in triplicate.

### 2.5. Cell Apoptosis Assay

*B7-H3* KO and mock H3255 and HCC827 cells were treated with CPT (8 *μ*M) for 6 h or gefitinib (0.05 *μ*M and 0.01 *μ*M for H3255 and HCC827, respectively) for 12 h. Annexin-V-APC and 7-aminoactinomycin D (7-AAD) double staining was conducted via an Annexin-V apoptosis kit (BD, Franklin Lakes, NJ) according to the manufacturer's instructions for adherent cells. Cells were analyzed by flow cytometry Epics XL-MCL (Beckman Coulter, CA, USA). Total apoptosis includes Annexin-V^+^/7-AAD^−^ (early apoptotic) and Annexin-V^+^/7-AAD^+^ (late apoptotic) cells.

### 2.6. Flow Cytometry Analysis

To verify gene deletion, *B7-H3* KO and mock H3255 and HCC827 cells were stained on ice with PE-conjugated human B7-H3 monoclonal antibody (Biolegend, San Diego, CA, USA) for 30 minutes. Cells were analyzed by flow cytometry on an Epics XL-MCL (Beckman Coulter) instrument. Data analysis and graphical output were performed using FlowJo (Version X; TreeStar, Ashland, OR, USA) software.

### 2.7. Western Blotting

*B7-H3* KO and mock H3255 and HCC827 cells were left untreated or treated with respective final concentrations of gefitinib for 24 h. The cell lysates were incubated with primary antibodies against human B7-H3 (1 : 500), total (t-) ERK1/2 (1 : 1000), phosphorylated (p-) ERK1/2 (T202+T204) (1 : 1000), t-STAT-3 (1 : 5000), *β*-actin (1 : 5000) (all from Abcam, Cambridge, MA, USA), t-Akt (1 : 1000), p-Akt (Ser473) (1 : 1000), and p-STAT-3 (Tyr705) (1 : 1000) (all from Affinity Biosciences, OH, USA). Next, horseradish peroxidase- (HRP-) conjugated mouse anti-rabbit IgG *κ* light chain binding protein (1 : 5,000, Santa Cruz Biotechnology, Santa Cruz, CA, USA) was exploited as secondary antibody. The membranes were then incubated with enhanced chemiluminescent (ECL) substrate (Thermo Fisher Scientific, Waltham, MA, USA) and visualized using the JS-1070PEV Fluorescent and Chemiluminescence Gel Imaging System (Peiqing, Shanghai, China). The expression level of signaling proteins was assessed using the ImageJ software, which is used to compare the density of bands on western blots.

### 2.8. Statistical Analysis

Experimental (*B7-H3* KO) groups were compared with mock controls treated or left untreated with gefitinib or CPT, where indicated. Results are means ± SEM of at least three representative experiments, where significance was calculated using two-tailed students *t*-test. The half maximal inhibitory concentration (IC_50_) values of gefitinib for H3255 and HCC827 cell lines were determined by exposing cells to an appropriate range of gefitinib concentrations, calculated using the Probit regression analysis by the SPSS software version 20.0 (SPSS, Chicago, IL, USA). ∗*P* < 0.05, ∗∗*P* < 0.01, and ∗∗∗*P* < 0.001 indicate the levels of significance.

## 3. Results

### 3.1. B7-H3 Ablation Reduces Proliferation of H3255 and HCC827 Strains

**Figure 1:**
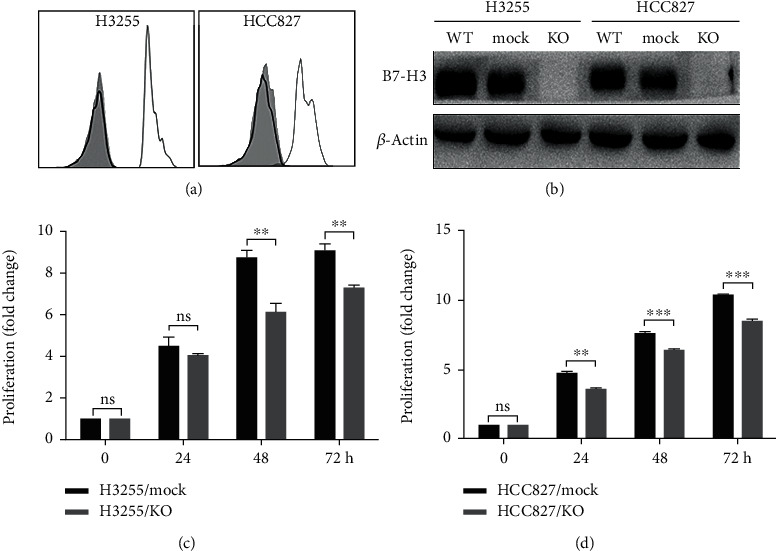
CRISPR/Cas9-mediated *B7-H3* KO reduces proliferation of H3255 and HCC827 strains. (a) Representative flow cytometry analysis for B7-H3 expression in H3255 KO and HCC827 KO cells. Grey shadow, IgG isotype control; grey and black lines correspond to mock and KO cells stained with anti-B7-H3, respectively. (b) Representative western blot analysis for B7-H3 expression in wild-type (WT), *B7-H3* KO, and mock H3255 and HCC827 cells as indicated. (c, d) The fold change proliferation of *B7-H3* KO and mock H3255 (c) and HCC827 (d) cells was determined by WST-8/CCK-8 at 0 h, 24 h, 48 h, and 72 h after cell culturing.

To explore B7-H3 effects on lung adenocarcinoma cells with EGFR mutation, we utilized CRISPR/Cas9 technology to delete *B7-H3* gene in H3255 and HCC827 cell lines. Both flow cytometry (Figure 1(a)) and western blotting analysis (Figure 1(b)) demonstrated that the two KO strains were devoid of B7-H3 protein. Yu et al. had shown that B7-H3 silencing reduced cell proliferation in EGFR wild-type A549 cells [17]. Herein we further demonstrate that *B7-H3* deletion also leads to substantially decreased proliferation of EGFR-mutated H3255 and HCC827 cells. The significant reduction of cell proliferation was observed for H3255 KO strains after time periods of 48 h and 72 h in culture, whereas for HCC827 KO cells at 24 h, 48 h, and 72 h after plating, respectively (Figures 1(c) and 1(d)).

### 3.2. B7-H3 KO Increases Apoptosis of H3255 and HCC827 Cells

**Figure 2:**
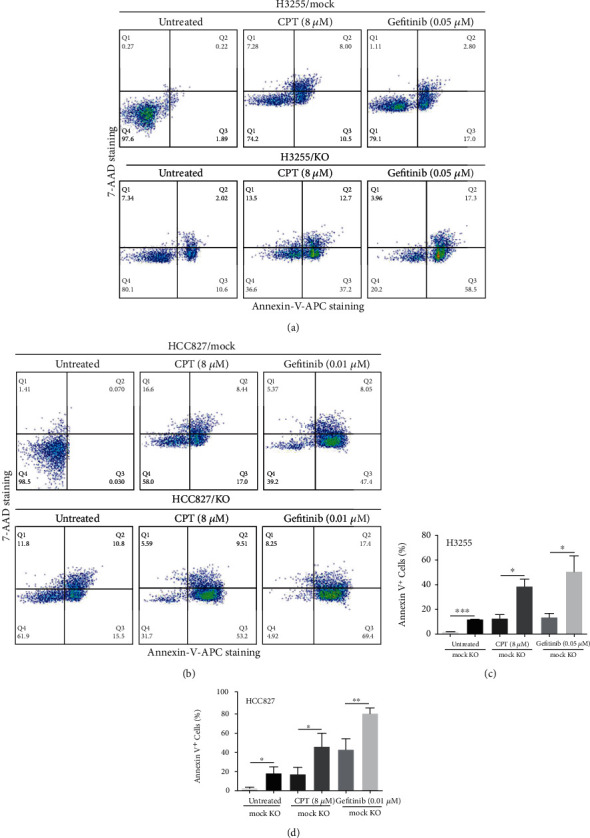
*B7-H3* deletion increases apoptosis of H3255 and HCC827 cells. *B7-H3* KO and mock H3255 (a) and HCC827 cells (b) were treated with 0.05 *μ*M and 0.01 *μ*M gefitinib, respectively, for 12 h or 8 *μ*M CPT for 6 h; cell apoptosis was analyzed by flow cytometry after Annexin-V/7-AAD double staining. All experiments were performed in triplicate, and the representative scatter plots are shown. (c, d) Comparison of the percentages of Annexin-V positive cells between *B7-H3* KO and mock cells.

To test the influence of *B7-H3* ablation on the spontaneous and induced apoptosis of EGFR-mutated lung adenocarcinoma cells, H3255 KO and HCC827 KO cells were left untreated or treated with 8 *μ*M CPT for 6 h and Annexin-V/7-AAD double staining was performed to determine cell apoptosis.  Figure 2 shows the flow cytometry profile for a representative experiment, which indicates 12.62% H3255 KO and 26.30% HCC827 KO cells were Annexin-V positive, compared to only 2.11% and 0.10% of corresponding mock cells, respectively (Figures 2(a) and 2(b)). Both the spontaneous apoptotic rates (%) of H3255 KO and HCC827 KO cells were significantly higher than the corresponding mock controls (*P* < 0.001 and *P* < 0.05) (Figures 2(c) and 2(d)). The total apoptotic rates (%) between CPT-induced *B7-H3* KO and mock H3255 cells were 38.79 ± 5.90 and 12.71 ± 3.17 (*P* < 0.05), and between CPT-induced *B7-H3* KO and mock HCC827 cells were 45.87 ± 8.37 and 16.91 ± 4.53 (*P* < 0.05), respectively (Figures 2(c) and 2(d)). Thus, *B7-H3* KO increases the apoptosis of lung adenocarcinoma cells with two EGFR mutant alleles Del E746-A750 and L858R.

### 3.3. B7-H3 KO Increases Gefitinib Susceptibility of H3255 and HCC827 Cells

**Figure 3:**
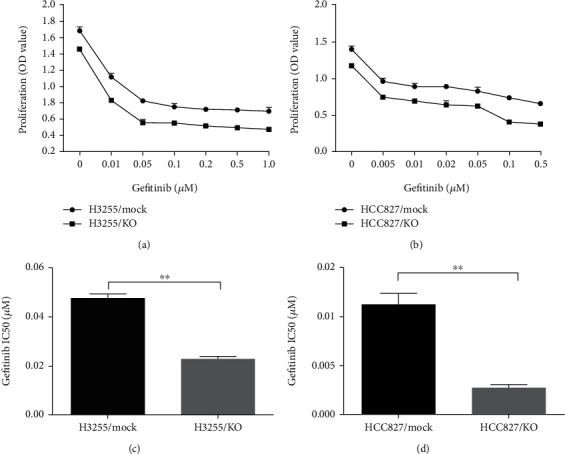
*B7-H3* deletion increases gefitinib susceptibility of H3255 and HCC827 cells. The concentration gradient of gefitinib between 0 and 1.0 *μ*M for H3255 KO and mock cells (a), and between 0 and 0.5 *μ*M for HCC827 KO and mock cells, (b) was added to the cell culture. 72 h later, the cell viability was determined by WST-8/CCK-8, and IC_50_ levels of H3255 KO/mock (c) and HCC827 KO/mock (d) cells were calculated using the Probit regression analysis by the SPSS software.

To explore the regulatory effects of B7-H3 on EGFR-TKIs treatment of lung adenocarcinoma harboring activated EGFR mutations, we administrated gefitinib to H3255 KO/mock and HCC827 KO/mock cell culture and monitored the ratio of apoptotic cells. The percentages of total apoptotic cells induced by gefitinib were 50.72 ± 12.91 and 13.62 ± 3.24 (%) (*P* < 0.05) for H3255 KO and mock cells, and 80.69 ± 3.47 and 43.07 ± 6.74 (%) (*P* < 0.01) for HCC827 KO and mock cells, respectively (Figures 2(a)–2(d)). On the other hand, Gefitinib's IC_50_ concentrations for H3255 KO and mock cells were 0.023 ± 0.012 and 0.044 ± 0.018 *μ*M (*P* < 0.01), and for HCC827 KO and mock cells were 0.003 ± 0.001 and 0.013 ± 0.003 *μ*M (*P* < 0.01), respectively (Figures 3(a)–3(d)). Thus, there are approximately 1-fold and 3-fold increment of the susceptibility of H3255 KO and HCC827 KO cells to gefitinib than the corresponding mock controls. Together, these results demonstrate that *B7-H3* ablation increases susceptibility of lung adenocarcinoma cells to EGFR-TKIs, especially in EGFR Del E746-A750-mutated HCC827 cells.

### 3.4. B7-H3-Induced Signaling in H3255 and HCC827 Cells

**Figure 4:**
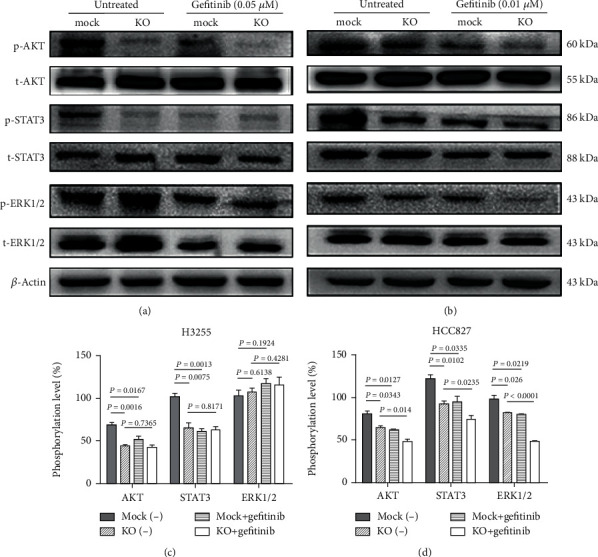
B7-H3-induced signaling in H3255 and HCC827 cells. *B7-H3* KO/mock H3255 (a) and HCC827 (b) cells were left untreated or treated with 0.05 *μ*M and 0.01 *μ*M gefitinib, respectively, and cell lysates were assayed for the expression of total and phosphorylated AKT, STAT3, and ERK1/2 by western blotting. These experiments were triplicated, and the representative images are shown. (c, d) Relative protein phosphorylation level ((p−/t−)∗100%) between groups. *β*-Actin was used as the reference protein. Data were mean ± SEM of three independent experiments.

Currently, the early events involved in B7-H3-induced signaling cascades are not known, and we detected total and phosphorylated AKT, STAT3, and ERK1/2 in *B7-H3* KO and mock H3255 and HCC827 cells treated or left untreated with gefitinib. As shown in Figure 4, both *B7-H3* deletion and gefitinib dramatically reduced the phosphorylation level of AKT and STAT3 in H3255 cells. *B7-H3* deletion is also similar with gefitinib in HCC827 cells, resulting in mild-to-moderate suppression on phosphorylation level of AKT, STAT3, and ERK1/2. However, significant synergistic effects were observed in HCC827 cells between *B7-H3* ablation and gefitinib (Figures 4(b) and 4(d)). Of note, both *B7-H3* deletion and gefitinib had no effects on the phosphorylation of ERK1/2 in H3255 cells (Figures 4(a) and 4(c)). Collectively, our results reveal difference of B7-H3-induced signaling between H3255 and HCC827 cells. We specifically address a translational potential of combined blockade of B7-H3-induced signaling and EGFR signaling in lung adenocarcinoma with EGFR Del E746-A750 mutation.

## 4. Discussion

In our experiments, the effects of B7-H3 are evident as *B7-H3* KO led to substantially reduced cell proliferation and increased apoptosis of H3255 and HCC827 strains. This result is consistent with previous observations of B7-H3 role in metastatic melanoma cells [19], multiple myeloma cells [20], and glioma cells [21], and also in line with the work of Yu et al. who showed that B7-H3 silencing strongly downregulates proliferation of EGFR wild-type A549 cells [17]. We extend their findings in EGFR-mutated lung adenocarcinoma cells and further demonstrate that *B7-H3* deletion increases the susceptibility of lung adenocarcinoma cells to gefitinib.

Our data demonstrate that PI3K/AKT, JAK2/STAT3, and Raf/MEK/ERK1/2 cascades are functional downstream B7-H3-induced signaling in HCC827 cells. However, only the PI3K/AKT and JAK2/STAT3 pathways are involved in B7-H3-induced signaling in H3255 cells. The inability of Raf/MEK/ERK1/2 cascade in H3255 cells is in agreement with previous study indicating that L858R mutation decreases ability to activate ERK1/2. The underlying mechanism may be related to reduced Y542 phosphorylation of SH2 domain-containing protein tyrosine phosphatase-2 (SHP-2), a tyrosine phosphatase required for the full activation of ERK [27]. The JAK2/STAT3 pathway is activated downstream B7-H3-induced signaling which is in agreement with reports of the B7-H3 role in multiple myeloma, hepatocellular carcinoma, and glioma cell lines [20–22]. On the other hand, B7-H3 was reported to induce drugs resistance and promote aerobic glycolysis through the PI3K/AKT pathway [23, 24]. Also, data from other groups showed that B7-H3 knockdown resulted in more sensitive of melanoma cells to small-molecule inhibitors targeting MAPK and AKT/mTOR pathways [19].

B7-H3-induced signaling and EGFR signaling at least partly share common downstream signaling cascades; we therefore put extensive efforts in identifying the potential correlation between the two pathways in H3255 and HCC827 cells. Our findings indicate that B7-H3-induced signaling and EGFR signaling have comparable effects on the activation of the PI3K/AKT, JAK2/STAT3, and Raf/MEK/ERK1/2 pathways both in H3255 and HCC827 cells. Both *B7-H3* deletion and gefitinib resulting in dramatic reduction of the phosphorylation level of AKT and STAT3 in H3255 cells while having mild-to-moderate suppression on phosphorylated AKT, STAT3, and ERK1/2 in HCC827 cells. However, *B7-H3* ablation had synergistic effects with gefitinib in HCC827 cells. These findings unlock a translational potential in guiding stratified therapeutic regimen for lung adenocarcinoma patients with EGFR Del E746-A750 mutation. Actually, gefitinib susceptibility of HCC827 KO cells was approximately 3-fold higher than HCC827 mock cells while that of H3255 KO cells was only 1-fold higher than the corresponding mock cells (Figures 3(c) and 3(d)). These results strongly suggest that B7-H3 and EGFR work in close collaboration to trigger downstream signaling cascades in H3255 cells. An interruption of either molecule is sufficient to suppress downstream signaling activities. However, these results imply that B7-H3 and EGFR work separately in triggering the common downstream signaling cascades in HCC827 cells. Thus, a combined blockade is essential for substantial inhibition of the signaling cascades.

It is to be noted that the possible way of B7-H3 to regulate PI3K/AKT, JAK2/STAT3, and Raf/MEK/ERK1/2 pathways remains to be elucidated in detail. B7-H3 is a type I transmembrane protein; however, its intracellular tail is short and has no known signaling motif [28]. Whether B7-H3-induced signaling is transduced by other transmembrane signaling molecules or intracellular adaptor proteins, which form heterodimers or polymers with B7-H3 totally unknown. Thus, further studies are needed, including our ongoing experiments, to comprehensively explore the underlying mechanisms of B7-H3-induced signaling in regulating the PI3K/AKT, JAK2/STAT3, and Raf/MEK/ERK1/2 pathways.

## 5. Conclusions

Our results contribute to the growing evidence that B7-H3-induced signaling promotes cell survival and reduces gefitinib sensitivity of lung adenocarcinoma cells with mutant EGFR alleles. Our findings give a clue that B7-H3-induced signaling is different in lung adenocarcinoma cells harboring EGFR L858R and Del E746-A750 mutations. Furthermore, our results strengthen the requirement for a combinative blockade in lung adenocarcinoma harboring EGFR Del E746-A750 mutation.

## Data Availability

The data used to support the findings of this study are available from the corresponding author upon request.
